# Increased Risk of Urticaria/Angioedema after BNT162b2 mRNA COVID-19 Vaccine in Health Care Workers Taking ACE Inhibitors

**DOI:** 10.3390/vaccines9091011

**Published:** 2021-09-11

**Authors:** Massimo Cugno, Dario Consonni, Andrea Lombardi, Patrizia Bono, Massimo Oggioni, Sara Uceda Renteria, Angela Cecilia Pesatori, Silvana Castaldi, Luciano Riboldi, Lorenzo Bordini, Carlo Domenico Nava, Ferruccio Ceriotti, Adriana Torri, Francesco Tafuri, Gabriele Ghigliazza, Flora Peyvandi, Alessandra Bandera, Andrea Gori

**Affiliations:** 1 Internal Medicine, Angelo Bianchi Bonomi Hemophilia and Thrombosis Center, Department of Pathophysiology and Transplantation, Università degli Studi di Milano, Fondazione IRCCS Ca’ Granda Ospedale Maggiore Policlinico, 20122 Milan, Italy; adriana.torri@fastwebnet.it (A.T.); francesco.tafuri@unimi.it (F.T.); gabriele.ghigliazza@unimi.it (G.G.); flora.peyvandi@unimi.it (F.P.); 2 Epidemiology Unit, Fondazione IRCCS Ca’ Granda Ospedale Maggiore Policlinico, 20122 Milan, Italy; dario.consonni@unimi.it (D.C.); angela.pesatori@unimi.it (A.C.P.); 3 Infectious Diseases Unit, Department of Pathophysiology and Transplantation, Università degli Studi di Milano, Fondazione IRCCS Ca’ Granda Ospedale Maggiore Policlinico, 20122 Milan, Italy; andrea.lombardi@unimi.it (A.L.); alessandra.bandera@unimi.it (A.B.); andrea.gori@unimi.it (A.G.); 4 Clinical Laboratory, Fondazione IRCCS Ca’ Granda Ospedale Maggiore Policlinico, 20122 Milan, Italy; patrizia.bono@policlinico.mi.it (P.B.); massimo.oggioni@policlinico.mi.it (M.O.); sara.ucedarenteria@policlinico.mi.it (S.U.R.); ferruccio.ceriotti@policlinico.mi.it (F.C.); 5 Department of Clinical Sciences and Community Health, Università degli Studi di Milano, 20122 Milan, Italy; 6 Quality Unit, Department of Biomedical Sciences for Health, Università degli Studi di Milano, Fondazione IRCCS Ca’ Granda Ospedale Maggiore Policlinico, 20122 Milan, Italy; silvana.castaldi@unimi.it; 7 Occupational Health Unit, Fondazione IRCCS Ca’ Granda Ospedale Maggiore Policlinico, 20122 Milan, Italy; luciano.riboldi@policlinico.mi.it (L.R.); lorenzo.bordini@policlinico.mi.it (L.B.); carlo.nava@policlinico.mi.it (C.D.N.); 8 Centre for Multidisciplinary Research in Health Science (MACH), Università degli Studi di Milano, 20122 Milan, Italy

**Keywords:** angioedema, urticaria, COVID-19 vaccination, mRNA vaccination, ACE inhibitors, heath care workers

## Abstract

Urticarial eruptions and angioedema are the most common cutaneous reactions in patients undergoing mRNA COVID-19 vaccinations. The vasoactive peptide bradykinin has long been known to be involved in angioedema and recently also in urticaria. Bradykinin is mainly catabolized by angiotensin-converting enzyme (ACE), which is inhibited by ACE inhibitors, a commonly employed class of antihypertensive drugs. We evaluated the risk of developing urticaria/angioedema after inoculation with the BNT162b2 mRNA COVID-19 vaccine in a population of 3586 health care workers. The influences of ACE inhibitors and selected potential confounding variables (sex, age, previous SARS-CoV-2 infection, and allergy history) were evaluated by fitting univariate and multivariable Poisson regression models. The overall cumulative incidence of urticaria/angioedema was 1.8% (65 out of 3586; 95% CI: 1.4–2.3%). Symptoms were mild, and no subject consulted a physician. Subjects taking ACE inhibitors had an adjusted three-fold increased risk of urticaria/angioedema (RR 2.98, 95% CI: 1.12–7.96). When we restricted the analysis to those aged 50 years or more, the adjusted RR was 3.98 (95% CI: 1.44–11.0). In conclusion, our data indicate that subjects taking ACE inhibitors have an increased risk of urticaria/angioedema after vaccination with the BNT162b2 mRNA COVID-19 vaccine. Symptoms are mild and self-limited; however, they should be considered to adequately advise subjects undergoing vaccination.

## 1. Introduction

After vaccination with the BNT162b2 mRNA COVID-19 vaccine, the main adverse events are short-term, mild-to-moderate pain at the injection site, fatigue, and headache [1]. However, cutaneous reactions have been described in subjects undergoing mRNA COVID-19 vaccinations and urticarial eruptions or angioedema were among the most common ones [2]. It has been known for more than twenty years that the increase in vascular permeability that leads to angioedema, in several cases, is due to the potent vasoactive peptide bradykinin [3,4,5] and recent data indicate that increased bradykinin production is not limited to patients with angioedema but also occurs in patients with urticaria [6]. Bradykinin is generated through two pathways: the first occurs by the action of cellular tissue kallikrein and the second by plasma kallikrein [7,8]. Concerning the first pathway, tissue kallikrein is produced by the cells of many organs and secreted as an active enzyme; it cleaves low-molecular-weight kininogen to release lys-bradykinin that is converted to bradykinin by an aminopeptidase [7]. The second pathway, which is present in plasma, leads to bradykinin generation through the cleavage of high-molecular-weight kininogen by plasma kallikrein during contact system activation [8,9]. The contact system consists of the substrate, high-molecular-weight kininogen, and the two zymogens, prekallikrein and factor XII, which activate each other in plasma to form the enzymes kallikrein and activated factor XII (FXIIa), respectively. In vitro, the system is activated after contact with negatively charged surfaces (hence the name) and the activation of prekallikrein to kallikrein depends on FXIIa [7,8,9,10]. In vivo, other pathways independent of FXIIa can also activate prekallikrein on endothelial cells, involving prolylcarboxypeptidase [11] and/or heat shock protein 90 [12]. The activation of the contact system with the generation of bradykinin is strictly linked to inflammation [13] and is also involved in mast cell-mediated allergic reactions [6,8,14]. The natural inhibitor of the system is the inhibitor of the first component of the complement; it is named C1-inhibitor and is able to block not only C1 but also FXIIa and kallikrein [15]. The hereditary and acquired deficiencies of C1-inhibitor are associated with angioedema syndromes due to hyperactivation of the contact system with generation of bradykinin [3,16]. Bradykinin causes vasodilation and increases vascular permeability by interacting with two types of receptors named B-1 and B-2. The B-2 receptors are constitutively expressed in vascular endothelial and smooth muscle cells whereas the B-1 receptors, which are expressed in the microcirculation only at a very low level, are induced by interleukin 1 (IL-1) and tumor necrosis factor-α (TNF-α) during inflammation [17]. The pharmacological block of B-2 receptors, obtained by the subcutaneous administration of the bradykinin receptor antagonist icatibant, is effective in reversing acute attacks of both hereditary [18] and acquired [19] angioedema. The degradation of bradykinin occurs by the action of several enzymes such as kininase I, named carboxypeptidase N, neutral endopeptidase, aminopeptidase P, and kininase II, which is the most important and is also known as angiotensin-converting enzyme (ACE) due to its effect on angiotensin [15,20]. Considering that bradykinin is mainly catabolized by ACE, subjects taking ACE inhibitors may have higher levels of bradykinin, leading to an increase in vascular permeability [21,22], especially in the presence of reduced levels in other catabolic enzymes [23,24]. ACE inhibitors are widely used, mainly in the treatment of hypertension and heart failure as well as in the prevention of macrovascular and microvascular disease associated with diabetes. In 2009, ACE inhibitors were the fourth most utilized drug class in the United States with 162.8 million prescriptions [25] and there were more than 24 million prescriptions of ramipril made in the UK in 2013 [26]. Thus, a lot of individuals taking ACE inhibitors may have an increased risk of urticaria/angioedema after COVID-19 vaccination. With this background, we performed a cohort study of vaccinated health care workers of a large university hospital in Milan, Italy to calculate the cumulative incidence (risk) of urticaria/angioedema after completion of BNT162b2 COVID-19 vaccination and the impact of ACE inhibitors on this risk.

## 2. Methods

In our hospital (Fondazione IRCCS Ca’ Granda Ospedale Maggiore Policlinico), one of the main hospitals in Milan, Lombardy, northwest Italy, the vaccination campaign with the BNT162b2 mRNA COVID-19 vaccine (Comirnaty, Pfizer-BioNTech) began on 27 December 2020. As per the vaccine schedule, the interval between the two doses was 21 days.

We performed a cohort study to calculate the cumulative incidence (risk) of urticaria/angioedema after completion of BNT162b2 COVID-19 vaccination and the impact of ACE inhibitors on this risk. The cohort included health care workers of the hospital vaccinated on 27 December 2020 or afterwards. One month after the second dose, health care workers were invited to fill in an acute reaction form at the time of blood collection to test for seroconversion by the assessment of anti-Spike-1 antibody values [27]. Demographics, vaccination data, and laboratory data were assembled by merging routinely collected databases. Clinical data collected with both vaccination and acute reaction questionnaires were stored in dedicated databases, as described in detail below.

The study was approved by the hospital’s ethics committee (Milano Area 2, Prot. No. 828_2021bis) and carried out in conformity with the 2013 revision of the Declaration of Helsinki. All subjects gave their written consent to participate in the study.

### 2.1. Cohort Description

The cohort database included the following occupations: physicians, nurses, midwives, health care assistants, health care technicians (biologists, radiology and laboratory assistants, psychologists and other health technicians), and clerical/administrative workers. Since it is a teaching hospital linked with the Università degli Studi di Milano, we also included students and residents in medicine and other health-related disciplines.

### 2.2. Vaccination Data

Our hospital coordinated three large vaccination centers in Milan (Policlinico, Fiera, and Scintille). Therefore, for subjects vaccinated within these three centers, the complete datasets containing demographics, dates of vaccination, and type of vaccine were directly available to us. For subjects vaccinated elsewhere, we completed information on vaccinations by linking the subjects in the cohort with a publicly available regional vaccination database. We used a deterministic linkage using the individual fiscal code as a unique identifier.

### 2.3. Laboratory Data

Data on previous SARS-CoV-2 infections were extracted from the laboratory databases, which contained results of real-time reverse transcriptase polymerase chain reaction (RT-PCR) tests performed on nasopharyngeal swabs (NPSs). Two different assays were used to detect SARS-CoV-2 RNA, *Allplex*™ SARS-CoV-2 assay (Seegene, Seoul, South Korea) on the CFX96 (Bio-Rad, Hercules, CA, USA) and the SARS-CoV-2 AMP Kit (Abbott, USA) on Alinity (Abbott, Chicago, IL, USA) in line with manufacturer’s instructions. The *Allplex*™ SARS-CoV-2 assay detects the SARS-CoV-2 N gene, E gene, and RdRp and S genes together. Cycle threshold (Ct) values were recorded for each gene and then averaged. Mean Ct values of less than 40 were interpreted as positive. The SARS-CoV-2 AMP Kit detects SARS-CoV-2 RdRp and N genes together; positive samples were those with Ct < 42.

### 2.4. Clinical Data

At the time of vaccination (either the first or second dose) each subject signed an informed consent form and filled in a short standardized questionnaire to investigate health status in the days before vaccination and history of allergy to vaccines and other substances as well as history of previous serious diseases and related therapies (e.g., anti-cancer, immunosuppression). The questionnaire was designed following a standard national format designed by the Ministry of Health. Data were stored in a dedicated Microsoft Access database.

### 2.5. Acute Reaction Data

A short structured questionnaire was designed and used to collect information about all types of reactions that occurred after vaccination schedule completion. As mentioned above, this questionnaire was administered at the time of blood collection to test for seroconversion by the assessment of anti-Spike-1 antibody values, about one month after the second vaccination dose. Data on acute reactions were stored in a dedicated Microsoft Access database.

### 2.6. Statistical Analysis

We merged and performed data editing of all the databases described above. We included in the analysis all cohort subjects satisfying the following criteria: vaccination with BNT162b2 (two doses); having filled in the vaccination form at the first dose and therefore having a record in the vaccination form database, which contains information on ACE inhibitor medication and on previous allergies; presentation for blood drawing and filling in the acute reaction questionnaire one month after the second dose.

In the overall cohort, we calculated the cumulative incidence (risk) of urticaria/angioedema and its 95% confidence interval (CI). Then, we examined the influence of ACE inhibitors and selected potential confounding variables, including sex, age class (<45, 45–49, 50–54, 55–59, and >60 years), previous SARS-CoV-2 infection, and allergy history), by fitting univariate and multivariable Poisson regression models with robust variance to calculate adjusted risk ratios (RRs) and 95% confidence intervals (CIs) [28,29]. Analyses were performed with Stata 17 (StataCorp 2021, College Station, TX, USA).

## 3. Results and Discussion

Between 18 February and 9 June 2021, 3586 health care workers filled in the acute reaction form at the time of blood drawing (one month after the second dose). There were 63 health care workers in therapy with ACE inhibitors, with similar distributions of sex and previous SARS-CoV-2 infection to the group not taking ACE inhibitors. Health care workers in therapy with ACE inhibitors were older (mean age 55.3 vs. 42.9 years), with almost 80% aged 50 years or more, and reported a more frequent history of allergy (33.3% vs. 20.5%) (Table 1).

The overall cumulative incidence of urticaria/angioedema was 1.8% (65 out of 3586; 95% CI: 1.4–2.3%). In univariate analyses, risk of urticaria/angioedema was higher in females and in subjects aged 45–49 years, was moderately increased in subjects with previous SARS-CoV-2 infection, and was strongly increased in subjects with allergy history and in those taking ACE inhibitors (Table 2). These findings were confirmed in multivariable analyses; in particular, persons taking ACE inhibitors had an adjusted three-fold increased risk of urticaria/angioedema. When we restricted the analysis to those aged 50 years or more, the adjusted RR was 3.98 (95% CI: 1.44–11.0).

In all subjects taking ACE inhibitors who reported urticaria/angioedema, urticaria/angioedema was defined as “light”. In all cases but one, urticaria/angioedema lasted less than one day and was not treated; only in one female, urticaria/angioedema lasted three days and required therapy with anti-histamine drugs. No one consulted a physician, went to an emergency department, or required hospitalization.

After considering potential confounding factors such as age, sex, previous SARS-CoV-2 infection and history of allergy, we found that the risk of developing urticaria/angioedema after vaccination with BNT162b2 in subjects taking ACE inhibitors was three times higher than that of subjects not taking ACE inhibitors, and four times higher when looking at subjects aged 50 years or more. Urticaria/angioedema reactions are generally minor and self-limited, and, according to several authors [2], they should not discourage vaccination. Although our data do not allow us to define the components of the vaccine that are implicated in the adverse events, the physiopathological basis of the higher risk of urticaria/angioedema in subjects taking ACE inhibitors may be the increase in bradykinin, a vasoactive peptide that induces vascular permeability and that is catabolized by ACE [21,22]. Indeed, in allergic reactions outside vaccination, the activation of the contact system (the mechanism generating bradykinin) has been demonstrated [14]. Theoretically, bradykinin may also worsen urticaria/angioedema due to allergies to vaccine components such as polyethylene glycol (PEG) [30]; however, no subjects of our study had a known allergy to PEG.

To the best of our knowledge, our study is the first investigating the association between ACE inhibitors and urticaria/angioedema after COVID-19 vaccination with an mRNA vaccine. The main limitations of the study were as follows: (1) not all health care workers responded to the invitation to perform a serology test; although this fact reduced the sample size, selection bias in reporting acute effects after vaccination is implausible, because participation was not associated with the development of adverse effects; (2) due to time constraints, we were not able to collect data on acute reactions after the first dose of vaccine; (3) our analysis was based on subjects’ self-reports and not on clinical diagnosis. Notwithstanding these limitations, we found an increased risk of urticarial/angioedema in persons taking ACE inhibitors which has a plausible physiopathological mechanism. Future studies on larger populations are needed to confirm and better characterize our findings.

In conclusion, in health care workers, urticaria/angioedema reactions are not frequent after vaccinations with the BNT162b2 mRNA COVID-19 vaccine; they are present in less than 2% of cases and increase to more than 6% in subjects taking ACE inhibitors. Although urticaria/angioedema symptoms are mild and self-limited, they should be taken into consideration by physicians in order to adequately advise subjects undergoing vaccination.

## Figures and Tables

**Table 1:** Demographic and clinical characteristics of health care workers included in the analysis of acute reactions after the second dose of COVID-19 vaccination with BNT162b2.

Variable	Subjects not Taking ACE Inhibitor Therapy N = 3523		Subjects Taking ACE Inhibitor Therapy N = 63		*p*-Value *
	N	%	N	%	
Gender					
Males	1008	28.6	21	33.3	0.41
Females	2515	71.4	42	66.7	
Age category (years)					
<45	1873	53.2	7	11.1	<0.001
45–49	415	11.8	6	9.5	
50–54	466	13.2	10	15.9	
55–59	420	11.9	24	38.1	
60+	349	9.9	16	25.4	
Previous SARS-CoV-2 infection	422	12.0	5	7.9	0.33
History of allergy	722	20.5	21	33.3	0.01

Abbreviations: ACE, angiotensin-converting enzyme. * *p*-values calculated with chi-squared test.

**Table 2:** Risk of urticaria/angioedema according to selected variables among health care workers included in the analysis of acute reactions after the second dose of COVID-19 vaccination with BNT162b2.

Variable	Urticaria/Angioedema		RR Crude		RR Adjusted *	
	N	%	RR	95% CI	RR	95% CI
Sex						
Male	11	1.1	1.00	Reference	1.00	Reference
Female	54	2.1	1.98	1.04–3.78	1.82	0.95–3.47
Age category (years)						
<45	24	1.3	1.00	Reference	1.00	Reference
45–49	13	3.1	2.42	1.24–4.71	2.24	1.15–4.35
50–54	10	2.1	1.65	0.79–3.42	1.53	0.74–3.15
55–59	9	2.0	1.59	0.74–3.39	1.35	0.63–2.85
60+	9	2.5	1.93	0.91–4.12	2.05	0.95–4.40
Previous SARS-CoV-2 infection						
No	54	1.7	1.00	Reference	1.00	Reference
Yes	11	2.6	1.51	0.80–2.88	1.63	0.87–3.06
History of allergy						
No	39	1.4	1.00	Reference	1.00	Reference
Yes	26	3.5	2.55	1.57–4.17	2.32	1.43–3.76
ACEI therapy						
No	61	1.7	1.00	Reference	1.00	Reference
Yes	4	6.4	3.68	1.38–9.80	2.98	1.12–7.96

Abbreviations: CI, confidence interval; RR, risk ratio (from Poisson regression models with robust variance). * Adjusted for all variables in the table.

## Data Availability

The datasets generated and/or analyzed during the current study are available from the corresponding author on reasonable request.
